# New Cytotoxic Secondary Metabolites from Marine Bryozoan *Cryptosula pallasiana*

**DOI:** 10.3390/md15040120

**Published:** 2017-04-13

**Authors:** Xiang-Rong Tian, Yan-Qing Gao, Xiao-Lin Tian, Jiao Li, Hai-Feng Tang, Yu-Shan Li, Hou-Wen Lin, Zhi-Qing Ma

**Affiliations:** 1 Research & Development Center of Biorational Pesticide, Northwest A&F University, Yangling 712100, China; tianxiangrong@163.com (X.-R.T.); gaoyanqinggc@nwsuaf.com.cn (Y.-Q.G.); 15591822363@163.com (X.-L.T.); 2 State Key Laboratory of Elemento-Organic Chemistry, College of Chemistry, Nankai University, Tianjin 300071, China; lijiao@mail.nankai.edu.cn; 3 Institute of Materia Medica, School of Pharmacy, Fourth Military Medical University, Xi’an 710032, China; 4 School of Traditional Chinese Medicines, Shenyang Pharmaceutical University, Shenyang 110016, China; lyshan888@126.com; 5 Department of Pharmacy, Renji Hospital, Affiliated to School of Medicine, Shanghai Jiao-Tong University, Shanghai 200127, China; franklin67@126.com

**Keywords:** marine bryozoan, *Cryptosula pallasiana*, sterol, ceramides, cytotoxicity

## Abstract

A new sterol, (23*R*)-methoxycholest-5,24-dien-3β-ol (**1**), two new ceramides, (2*S*,3*R*,4*E*,8*E*)-2-(tetradecanoylamino)-4,8-octadecadien-l,3-diol (**6**) and (2*S*,3*R*,2′*R*,4*E*,8*E*)-2-(tetradecanoylamino)-4,8-octadecadien-l,3,2′-triol (**7**), together with three known sterols (**2**–**4**), a lactone (**5**) and two ceramides (**8**,**9**), were isolated from the marine bryozoan *Cryptosula pallasiana*, collected at Huang Island of China. The structures of the new compounds were elucidated by extensive spectroscopic analyses, chemical methods and quantum electronic circular dichroism (ECD) calculations. Among the isolated compounds, sterol **1** possessed a rare side chain with a methoxy group at C-23, and a double bond between C-24 and C-25. Ceramides **6** and **7** possessed 14 carbons in their long-chain fatty acid base (FAB), which were different from the normal ceramides with 16 carbons in the FAB. Moreover, compounds **5** and **8** were isolated for the first time from marine bryozoans. Compounds **1**–**9** were evaluated for their cytotoxicity against human tumor cell lines HL-60, Hep-G2 and SGC-7901. The results showed that lactone **5** appears to have strong cytotoxicity against the test tumor cell lines, with IC_50_ values from 4.12 μM to 7.32 μM, and sterol **1** displayed moderate cytotoxicity with IC_50_ values between 12.34 μM and 18.37 μM, while ceramides **6**–**9** showed weak cytotoxicity with IC_50_ ranging from 21.13 μM to 58.15 μM.

## 1. Introduction

**Figure 1:**
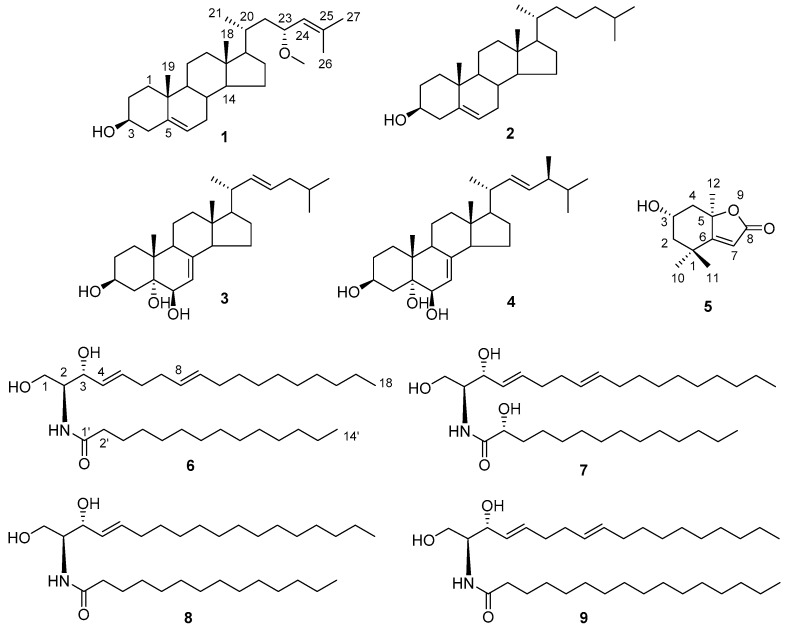
Structures of compounds **1**–**9** from marine bryozoan *C. pallasiana*.

Marine bryozoans are important sources for the discovery of new bioactive secondary metabolites. There are three reports containing 19 new metabolites isolated from this understudied phylum in the last year alone [1]. Modern phytochemical and pharmacological investigations of marine bryozoans demonstrated that secondary metabolites, including macrolides, alkaloids, sterols, as well as halogen-containing compounds, etc., exhibited remarkable cytotoxic activities on tumor cell lines, such as human myeloid leukemia HL-60, human leukemia U937, murine lymphocytic leukemia P388, and so on [2,3]. *Cryptosula pallasiana* is an encrusting colonial marine bryozoan, belonging to the class Gymnolaemata, order Cheilostomata and family Cryptosulidae. In our previous investigation on the bioactive compounds from this marine bryozoan, 16 alkaloids, 13 sterols, three aromatic compounds and two glycerol derivatives have been identified [4,5,6]. In the course of our ongoing discovery of new biologically active secondary metabolites from marine bryozoans, *C. pallasiana,* collected from the coasts of Huang Island in Qingdao City, Shandong Province of China, was investigated. We report herein the isolation and structure elucidation of three new compounds including one sterol (**1**) and two ceramides (**6** and **7**), along with six known secondary metabolites including three sterols (**2**–**4**), one lactone (**5**) and two ceramides (**8**,**9**). The structures of the known compounds were identified by comparison of their physical and spectroscopic data with those reported in the literature (Figure 1). In addition, the cytotoxicity of the isolated compounds against human tumor cell lines HL-60, Hep-G2 and SGC-7901 are also described.

## 2. Results

The 95% EtOH extract of *C. pallasiana* was concentrated and partitioned with petroleum ether, CCl_4_ and *n*-BuOH, respectively. The resulting CCl_4_ portion was separated by column chromatography (CC) and high-performance liquid chromatography (HPLC) to afford a new sterol (**1**) and two new ceramides (**6** and **7**), along with six known compounds (**2**–**5**, **8** and **9**) (Figure 1). The known compounds were identified as cholest-5-en-3β-ol (**2**) [7], (22*E*)-cholesta-7,22-dien-3β,5α,6β-triol (**3**) [8], (24*S*,22*E*)-methylcholesta-7,22-dien-3β,5α,6β-triol (**4**) [8], loliolide (**5**) [9], (2*S*,3*R*,4*E*)-2-(tetradecanoylamino)-4-octadecen-l,3-diol (**8**) [10] and (2*S*,3*R*,4*E*,8*E*)-2-(hexadecanoylamino)-4,8-octadecadien-l,3-diol (**9**) [11], respectively, by comparison of their spectroscopic data (see supplementary information) with those reported in the literature.

**Figure 2:**
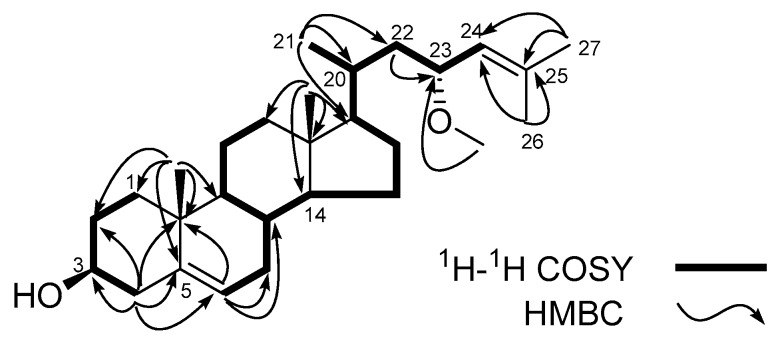
Key ^1^H-^1^H COSY and HMBC correlations of **1**.

Compound **1** was obtained as a white amorphous powder. Its molecular formula was determined as C_28_H_46_O_2_ on the basis of the positive high-resolution electrospray ionization mass spectroscopy (HRESIMS) *m/z* 437.3395 [M + Na]^+^ (C_28_H_46_O_2_Na, calcd. for 437.3396). The ^1^H-NMR spectrum of **1** showed two tertiary methyl resonance signals at δ_H_ 0.73, s (H_3_-18) and 1.00, s (H_3_-19), three secondary methyl groups at δ_H_ 0.95, d, *J* = 5.9 Hz (H_3_-21), δ_H_ 1.67, d, *J* = 1.0 Hz (H_3_-26) and 1.74, d, *J* = 1.1 Hz (H_3_-27), one methoxy group at δ_H_ 3.21 s (OMe-23), two oxymethine protons at δ_H_ 3.52, m, (H-3) and 3.94, dt, *J* = 9.3, 3.1 Hz (H-23), and two tri-substituted olefinic protons at δ_H_ 5.35, t, *J* = 2.5 Hz (H-6) and 5.03, td, *J* = 9.0, 2.6, 1.3 Hz (H-24). The ^13^C-NMR spectrum showed 28 carbon resonances, which were assigned by distortionless enhancement by polarization transfer (DEPT) and heteronuclear single quantum coherence (HSQC) spectra to one methoxy carbon at δ_C_ 55.7 (OMe-23), five methyl (C-18, C-19, C-21, C-26 and C-27), nine methylene sp^3^ (C-1, C-2, C-4, C-7, C-11, C-12, C-15, C-16 and C-22), seven methine sp^3^ (C-3, C-8, C-9, C-14, C-17, C-20 and C-23) including two hydroxymethines C-3 (δ_C_ 71.8) and C-23 (δ_C_ 74.8), two quaternary sp^3^ carbons (C-10 and C-13), two methine sp^2^ carbons C-6 (δ_C_ 121.7) and C-24 (δ_C_ 127.0), and two quaternary sp^2^ carbons C-5 (δ_C_ 140.7) and C-25 (δ_C_ 134.5). These data suggested that **1** possessed a 3-hydroxy ∆^5^-steroid nucleus with a methoxy group and double bond in its side chain. Further structural information for compound **1** was obtained by 2D NMR analysis including ^1^H-^1^H correlation spectroscopy (COSY) and heteronuclear multiple bond correlation (HMBC) spectra (Figure 2). The observation of correlations from H-23 to H_2_-22 and H-24 in the ^1^H-^1^H COSY spectrum, and correlations from methoxy proton to C-23, from H_2_-22 to C-23, from H_3_-26 to C-24 and C-25, and from H_3_-27 to C-24 and C-25 in the HMBC spectrum indicated that the methoxy group is on C-23, and the tri-substituted double bond is between C-24 and C-25. Based on the above evidences, the planar structure of sterol **1** could be established as 23-methoxycholest-5,24-dien-3-ol. 

**Figure 3:**
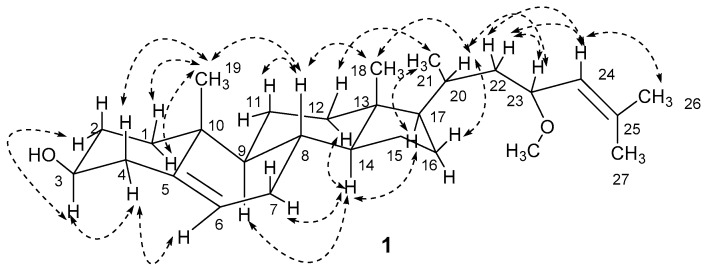
Key NOESY correlations (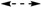) for **1**.

**Figure 4:**
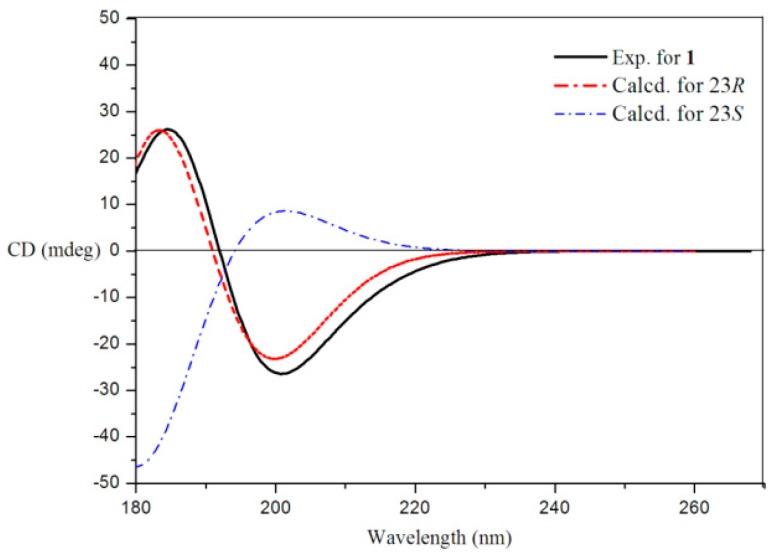
Experimental and calculated ECD spectra for **1**.

The stereochemistry of **1** was established by extensive analysis of nuclear overhauser effect spectroscopy (NOESY) spectrum and quantum electronic circular dichroism (ECD) calculations*.* The ^1^H-NMR assignment of α and β protons for compound **1** (Table 1) was based on careful analysis of the NOESY spectrum as well as comparison with the reference [5] (Figure 3). The NOESY correlations from H_3_-19 to H-1α and H-1β, from H-3 to H-4α and H-2α, and from H-4α to H-6, suggested that the A-ring exists in a chair conformation. The 3-hydroxyl group was oriented in an equatorial position and therefore in a β-orientation, which was confirmed by the chemical shift of C-3 (δ_C_ 71.8 > 70.0) [12]. Similarly, the presence of a cross-peak between H-14 and H-17 implied that the C-17 side chain was in a β-position. The correlations from H_3_-21 to H-12β and H-17, and from H-20 to H-16β and H_3_-18 indicating the α-orientation of the methyl group at C-20, and determined the C-20*S* configuration for **1**. The C-23*R* configuration was determined by ECD calculations of 23*R* and 23*S* for **1** using the time-dependent density functional theory (TD-DFT) method at the B3LYP/6-31G (d) level [13,14,15,16]. The preliminary conformational distribution search was performed by HyperChem 7.5 software (Hypercube, Inc., Gainesville, FL, USA). The corresponding minimum geometries were fully optimized by using DFT at the B3LYP/6-31G (d) levels implemented in the Gaussian 09 program package. The ECD calculations were performed after optimization of the selected conformers at the B3LYP/6-31G (d) [17,18]. The result showed that the experimental circular dichroism (CD) curve of **1** was consistent with the calculated ECD curve of 23*R*, while opposite to that of 23*S* (Figure 4). The NOESY correlations from H-23 to H-20, H-22α and H_3_-26, and from H-24 to H-22α, H-22β and H_3_-27, indicated the α-orientation of the methoxy group at C-23, which also confirmed the 23*R* configuration. Thus, sterol **1** was unambiguously elucidated as (23*R*)-methoxycholest-5,24-dien-3β-ol.

**Figure 5:**
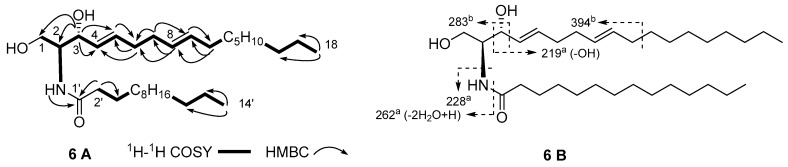
Key ^1^H-^1^H COSY and HMBC correlations (**A**), and main ESI-MS fragment ions (**B**) of **6**. (^a^ positive fragment ions; ^b^ negative fragment ions).

Compound **6** was obtained as a white amorphous powder. The (+)-HRESIMS showed a pseudomolecular ion peak at *m/z* 530.4545 [M + Na]^+^ (calcd. for C_32_H_61_NO_3_Na, 530.4549), which, together with the pseudomolecular ion peak at *m/z* 506 [M − H]^−^ in the (‒)-ESIMS spectrum, enabled the determination of the molecular formula for **6** as C_32_H_61_NO_3_. A careful analysis of the ^1^H- and ^13^C-NMR spectra of **6** (Table 2) by DEPT and HSQC experiments revealed the presence of an amide group δ_C_ 174.0 (C-1′), δ_H_ 6.25, d, *J* = 7.5 Hz (NH), a nitrogen- or oxygen-bearing methine δ_C_ 54.5 (C-2), δ_H_ 3.95, dd, *J* = 11.3, 3.5 Hz (H-2), an oxymethylene sp^3^ δ_C_ 62.5 (C-1), δ_H_ 3.70, dd, *J* = 11.3, 3.3 Hz and 3.90, m (H_2_-1), an oxymethine sp^3^ δ_C_ 74.7 (C-3), δ_H_ 4.32, *m* (H-3), and four methine sp^2^ δ_C_ 129.2 (C-4), δ_H_ 5.54, dd, *J* = 15.4, 6.4 Hz (H-4); δ_C_ 133.6 (C-5), δ_H_ 5.78, dt, *J* = 15.4, 6.6 Hz (H-5); δ_C_ 131.4 (C-8), δ_H_ 5.42, dt, *J* = 15.2, 6.3 Hz (H-8) and δ_C_ 129.0 (C-9), δ_H_ 5.37, dt, *J* = 15.2, 6.4 Hz (H-9), which together with a series of alkyl proton signals (δ_C_ 29.2–29.7, δ_H_ 1.26, *br s*), indicated an existence of a sphinga-4,8-diene skeleton for **6** [19]. The ^1^H-^1^H COSY correlations from H-2 to NH, H_2_-1 and H-3, and the HMBC correlations from NH to C-1′, from H_2_-2′ to C-1′ and C-3′, and from H-3 to C-1 and C-2 confirmed the sphingosine skeleton. That the two double bonds were placed on C-4 and C-8 was supported by ^1^H-^1^H COSY correlations of H-3/H-4, H-4/H-5, H-5/H_2_-6, H_2_-6/H_2_-7, H_2_-7/H-8, H-8/H-9 and H-9/H_2_-10, as well as by HMBC correlations from H-3 to C-4 and C-5, from H-8 to C-6 and C-7, and from H_2_-10 to C-8 and C-9 (Figure 5A). That the sphingoid long chain base (LCB) and the long chain fatty acid base (FAB) of the amide portion contained 18 and 14 carbons, respectively, was evidenced by the electrospray ionization mass spectroscopy (ESI-MS) fragments at *m/z* 219, 228, 262, 283, 394 (Figure 5B) [20]. The number of the carbon for the FAB was further validated through electron impact mass spectrometry (EI-MS) molecular ion at *m/z* 242 [M]^+^ for the methyl tetradecanoate obtained after methanolysis of **6** with 5% HCl-MeOH. Therefore, the planar structure and the key connectivities of **6** were established.

That the geometry of C-4/C-5 and C-8/C-9 double bonds are *trans* was based on the vicinal coupling constants *J*_4,5_ = 15.4 Hz and *J*_8,9_ = 15.2 Hz, as well as the chemical shifts of C-6 (δ_C_ 32.3) and C-7 (δ_C_ 32.1) [21]. The absolute stereochemistry of **6** was determined as d-(*−*)-*erythro*-2*S*,3*R* configuration based on the chemical shifts of C-2 (δ_C_ 54.5) and C-3 (δ_C_ 74.7), which were in good agreement with those reported for *N*-palmitoyl-d-*erythro*-(2*S*,3*R*)-octadecasphinga-4(*E*)-en (C-2 for 54.5 and C-3 for 74.7) [22,23]. Based on the above analyses, the structure of **6** was determined as (2*S*,3*R*,4*E*,8*E*)-2-(tetradecanoylamino)-4,8-octadecadien-l,3-diol.

**Figure 6:**
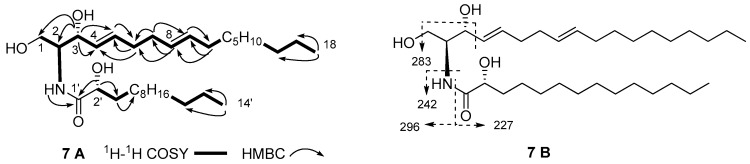
Key ^1^H-^1^H COSY and HMBC correlations (**A**), and main ESI-MS fragments (**B**) of **7**.

The molecular formula of compound **7** was assigned as C_32_H_61_NO_4_ on the basis of (+)-HRESIMS pseudomolecular ion peak at *m/z* 546.4496 [M]^+^ (calcd. for C_32_H_61_NO_4_Na, 546.4498) and the ^1^H- and ^13^C-NMR spectra (Table 2). The ^13^C-NMR spectrum of **7** showed a close resemblance to that of **6**, except for the additional hydroxyl group on C-2′ in the FAB, since **7** showed different chemical shifts of C-1′ (δ_C_ 175.0), C-2′ (δ_C_ 72.4) and C-3′ (δ_C_ 34.8) in its FAB. HMBC correlations of H-2′ (δ_H_ 4.12) to C-1′ and C-3′ confirmed that the hydroxyl group was on C-2′ (Figure 6A). A series of fragment ions at *m/z* 227, 242, 283 and 296 in the (‒)-ESIMS spectrum (Figure 6B), deduced the lengths of LCB and FAB to be 18 and 14 carbons, respectively. Additionally, the length of FAB was further validated through (+)-EIMS molecular ion at *m/z* 258 [M]^+^ of (*R*)-methyl 2-hydroxytetradecanoate obtained from methanolysis of **7**. Comparisons of the optical rotation of **7** (${\left[\alpha \right]}_{D}^{22}$ +9.5°) with (2*S*,3*R*,2′*R*,4*E*)-2′-hydroxy-C16-ceramide ([α]_D_ +5.2°) and its 2′*S*-isomer ([α]_D_ −16.1°), suggested that **7** and (2*S*,3*R*,2′*R*,4*E*)-2′-hydroxy-C16-ceramide have the same absolute configurations for core chiral centers C-2, C-3 and C-2′ [24]. Thus, compound **7** was determined as (2*S*,3*R*,2′*R*,4*E*,8*E*)-2-(tetradecanoylamino)-4,8-octadecadien-l,3,2′-triol.

Compounds **1**–**9** were evaluated for their cytotoxicity against human myeloid leukemia cell line HL-60, human hepatocellular carcinoma HepG-2, and human gastric carcinoma SGC-7901 using an MTT assay, and results are shown in Table 3. Compound **1** displayed a moderate cytotoxicity to the test cell lines with IC_50_ values between 12.34 μM and 18.37 μM, while **2**–**4** did not show any cytotoxicity. Since **5** exhibited strong cytotoxicity against the test tumor cell lines with IC_50_ values between 4.12 μM and 7.32 μM, it can be considered as a potential for the further development of an anticancer agent. Compounds **6**–**9** showed weak cytotoxicity against all the test tumor cell lines with IC_50_ values ranging from 21.13 μM to 58.15 μM; however, they showed stronger cytotoxicity against HepG-2 and SGC-7901 than HL-60, suggesting a selectivity of these ceramides against HepG-2 and SGC-7901. Furthermore, ceramide **7,** having the 2′-OH group in its FAB, displayed stronger cytotoxicity than its dehydroxy counterparts, indicating that the 2′-OH group in the FAB may play an important role in the expression of cytotoxicity for ceramides [24,25].

## 3. Experimental Section

### 3.1. General Experimental Procedures

Optical rotations were measured on a Perkin-Elmer 343 polarimeter. 1D and 2D NMR spectra were obtained in CDCl_3_ or CD_3_OD on a Bruker AVANCE-500 spectrometer, with tetramethylsilane (TMS) as an internal standard. Chemical shifts (δ) were expressed in ppm and coupling constants in Hz. EI-MS spectrum was obtained on a Finnigan MAT 212 mass spectrometer. ESI-MS and HR-ESI-MS spectra were taken on a Micromass Quattro mass spectrometer (Micromass, Manchester, UK). CD spectrum was recorded on JASCOJ-720 spectrophotometer (JASCO Corporation, Tokyo, Japan). Separation and purification were performed by CC on silica gel H (10–40 μm, Qingdao Marine Chemical Inc., Qingdao, China), Sephadex LH-20 (Pharmacia Inc., New Jersey, NJ, USA) and reversed-phase Si gel (Lichroprep RP-18, 40–63 μm, Merck Inc., Darmstadt, Germany). Semi-preparative HPLC was carried out on a Dionex P680 liquid chromatograph equipped with a UV 170 UV/Vis detector at 206 nm using a YMC-Pack R&D ODS-A column (250 mm × 20 mm i.d., 5 μm, YMC, Kyoto, Japan). Compounds detection in the thin-layer chromatography (TLC) plate was achieved by spraying the silica gel plates (Qingdao Marine Chemical Inc., Qingdao, China) with 15% H_2_SO_4_ in ethanol followed by heating.

### 3.2. Animal Material

The marine bryozoan *Cryptosula pallasiana* was collected in March 2009 from Huang Island, Qingdao City, Shandong Province of China, was identified by one of us (H.-W. Lin). A voucher specimen (No: QD-0903-1) was deposited in Marine Laboratory, Changzheng Hospital, Second Military Medical University.

### 3.3. Extraction and Isolation

The fresh animal *C. pallasiana* (about 20 kg) was exhaustively extracted with 95% EtOH at room temperature. Followed the extraction procedure we described previously, CCl_4_ fraction (about 12.9 g) was further separated by CC on Sephadex LH-20 with CHCl_3_/MeOH (1:1) as an eluting solvent to afford three fractions (Frs. A–C) [5]. Fr. A (5.56 g) was subjected to a reversed-phase silica gel column eluting with a mixture of MeOH/H_2_O (4:1) and MeOH to afford Fr. A_1_ and Fr. A_2_, respectively. Fr. A_2_ was submitted to a silica gel column and eluted with petroleum ether/EtOAc (15:1, 10:1, 5:1, 1:1) to give 12 fractions (Frs. A_2_-1–A_2_-12). The isolation of 16 alkaloids, 13 sterols, three aromatic compounds and two glycerol derivatives from Fr. B, Fr. C, and Fr. A_2_-4 to Fr. A_2_-9 have been reported previously [4,5,6]. Fr. A_2_-3 (183.0 mg) was purified by semi-preparative HPLC to give **1** (2.5 mg, *t*_R_ = 67.2 min) and **2** (20.9 mg, *t*_R_ = 73.4 min), using MeOH/H_2_O (19:1) as mobile phase at a flow rate of 8.0 mL/min. Fr. A_2_-11 (114.7 mg) was purified by semi-preparative HPLC to obtain **3** (6.2 mg, *t*_R_ = 47.7 min) and **4** (20.9 mg, *t*_R_ = 54.4 min), using MeOH/H_2_O (9:1) as mobile phase at a flow rate of 8.0 mL/min. Fr. A_1_ (2.4 g) was eluted with CHCl_3_/MeOH (1:1) on Sephadex LH-20, and then further purified by semi-preparative HPLC to give **5** (4.0 mg, *t*_R_ = 39.6 min), using MeOH/H_2_O (1:3) as mobile phase at a flow rate of 8.0 mL/min. Fr. A_2_-10 (150.0 mg) was eluted with CHCl_3_/MeOH (1:1) on Sephadex LH-20, and then further purified by semi-preparative HPLC to afford **6** (9.3 mg, *t*_R_ = 49.5 min), **7** (7.4 mg, *t*_R_ = 46.3 min), **8** (8.5 mg, *t*_R_ = 51.4 min) and **9** (7.1 mg, *t*_R_ = 54.7 min), using MeOH/H_2_O (19:1) as mobile phase at a flow rate of 8.0 mL/min.

#### 3.3.1. (23*R*)-Methoxycholest-5,24-dien-3β-ol (**1**)

White amorphous powder, ${\left[\alpha \right]}_{D}^{22}$ −22.0° (*c* 0.05, CHCl_3_); ^1^H- and ^13^C-NMR data see Table 1; (+)-ESI-MS *m/z*: 437 [M + Na]^+^, 415 [M + H]^+^, 385, 349, 318, 274, 256; (+)-HRESIMS *m/z*: 437.3395 [M + Na]^+^ (calcd. for C_28_H_46_O_2_Na, 437.3396).

#### 3.3.2. (2*S*,3*R*,4*E*,8*E*)-2-(Tetradecanoylamino)-4,8-octadecadien-l,3-diol (**6**)

White amorphous powder, ${\left[\alpha \right]}_{D}^{22}$ −5.7° (*c* 0.10, CHCl_3_); ^1^H- and ^13^C-NMR data see Table 2; (+)-ESIMS *m/z*: 530 [M + Na]^+^, 437, 330, 302, 274, 262, 256, 228, 219; (‒)-ESIMS *m/z*: 552 [M + COOH]^−^, 542 [M + Cl]^−^, 506 [M − H]^−^, 447, 394, 341, 283, 255, 218, 186, 143, 126; (+)-HRESIMS *m/z*: 530.4545 [M + Na]^+^ (calcd. for C_32_H_61_NO_3_Na, 530.4549).

#### 3.3.3. (2*S*,3*R*,2′*R*,4*E*,8*E*)-2-(Tetradecanoylamino)-4,8-octadecadien-l,3,2′-triol (**7**)

White amorphous powder, ${\left[\alpha \right]}_{D}^{22}$ +9.5° (*c* 0.10, CHCl_3_); ^1^H- and ^13^C-NMR data see Table 2; (+)-ESIMS *m/z*: 546 [M + Na]^+^, 437, 330, 274, 262, 256; (‒)-ESIMS *m/z*: 558 [M + Cl]^−^, 522 [M − H]^−^, 340, 296, 283, 242, 227; (+)-HRESIMS *m/z*: 546.4496 [M + Na]^+^ (calcd. for C_32_H_61_NO_4_Na, 546.4498).

### 3.4. Methanolysis of Compounds ***6*** and ***7***

Compounds **6** and **7** (each about 3 mg) were dissolved in 5% HCl-MeOH (3 mL), respectively, and then refluxed for 12 h at 80 °C. The reaction mixture was extracted with *n*-hexane (3 × 4 mL). The *n*-hexane portion was washed with H_2_O and concentrated in vacuo to yield methyl tetradecanoate and (*R*)-methyl 2-hydroxytetradecanoate, respectively. The H_2_O portion was neutralized with NH_4_OH, and then further concentrated in vacuo to yield the corresponding LCB with specific rotation ${\left[\alpha \right]}_{D}^{22}$ at −3.4° (c 0.05, CHCl_3_).

Methyl tetradecanoate: EI-MS *m/z* 242 [M]^+^ (25), 211 [M − CH_3_]^+^ (11), 199 [M − COCH_3_]^+^ (22), 185 (8), 171 (4), 157 (7), 143 (22), 129 (9), 115 (4), 101 (9), 87 (61), 74 (100), 55 (24).

(*R*)-Methyl 2-hydroxytetradecanoate: ${\left[\alpha \right]}_{D}^{22}$ −6.5° (*c* 0.05, CHCl_3_); EI-MS *m/z* 258 [M]^+^ (7), 199 [M − COCH_3_]^+^ (65), 125 (30), 111 (56), 90 (47), 83 (85), 69 (100), 55 (92).

### 3.5. MTT Cytotoxicity Assays

Compounds **1**–**9** were evaluated for their cytotoxic activity against human tumor cell lines HL-60, HepG-2 and SGC-7901 using MTT assay. The cell lines were obtained from American type culture collection (ATCC), and were seeded to RPMI-1640 medium with 10% fetal bovine serum and 100 U/mL benzyl penicillin-streptomycin solutions, at 37 °C in a humidified atmosphere with 5% CO_2_/air for 24 h. Then the test samples were added and incubated at 37 °C for another 72 h. The detailed procedure for MTT assay can be found in our previous published literature [5]. The cytotoxic activity was expressed as IC_50_ value using adriamycin as a positive control. 

## 4. Conclusions

A new sterol (**1**), two new ceramides (**6**,**7**) and six known compounds (**2**–**5**,**8**,**9**) were identified from the marine bryozoan *C. pallasiana*. Among the isolated compounds, sterol **1** showed novelty in the C-23*R* methoxy group and double bond between C-24 and C-25 in its side chain. Since this is the first report of ceramides in this species, it can be considered important for its chemotaxonomic significance. Furthermore, ceramides with 14 carbons in the FAB were different from those we previously obtained from *Bugula neritina* [25,26]. All compounds were evaluated for their cytotoxicity, and lactone (**5**) was found to exhibit stronger cytotoxicity than sterols and ceramides, suggesting that it could be responsible for the cytotoxicity of *C. pallasiana*.

## Figures and Tables

**Table 1:** ^1^H- and ^13^C-NMR (500 and 125 MHz, CDCl_3_) data of **1** ^a^.

No.	δ_C_	δ_H_ mult., *J* in Hz	No.	δ_C_	δ_H_ mult., *J* in Hz
1	37.3	α 1.86, m	13	42.5	–
		β 1.08, m	14	56.9	1.04, m
2	31.7	α 1.84, m	15	24.3	α 1.57, m
		β 1.50, m			β 1.07, m
3	71.8	3.52, m	16	28.2	α 1.83, m
4	42.3	α 2.28, m			β 1.30, m
		β 2.23, m	17	56.8	1.01, m
5	140.7	–	18	12.0	0.73, s
6	121.7	5.35, t (2.5)	19	19.4	1.00, s
7	31.9	α 1.50, m	20	32.5	1.66, m
		β 1.97, m	21	18.7	0.95, d (5.9)
8	31.9	1.50, m	22	42.5	α 0.92, m
					β 1.68, m
9	50.1	0.93, m	23	74.8	3.94, dt (9.3, 3.1)
10	36.5	–	24	127.0	5.03, td (9.0, 2.6, 1.3)
11	21.1	α 1.43, m	25	134.5	–
		β 1.49, m	26	18.2	1.67, d (1.0)
12	39.8	α 1.16, m	27	25.9	1.74, d (1.1)
		β 2.03, m	23-OCH_3_	55.7	3.21, s

^a^ Assignments based on DEPTs and HSQC.

**Table 2:** ^1^H- and ^13^C-NMR (500 and 125 MHz, CDCl_3_) of **6** and **7** ^a^.

No.	6		7	
	δ_C_	δ_H_ mult., *J* in Hz	δ_C_	δ_H_ mult., *J* in Hz
1	62.5	3.70, dd (11.3, 3.3)	62.2	3.72, dd (11.0, 3.0)
		3.90, m		3.89, m
2	54.5	3.95, dd (11.3, 3.5)	54.4	3.92, br d (11.0)
3	74.7	4.32, m	74.7	4.30, m
4	129.2	5.54, dd (15.4, 6.4)	129.2	5.52, dd (15.5, 6.0)
5	133.6	5.78, dt (15.4, 6.6)	133.5	5.75, dt (15.5, 6.1)
6	32.3	1.97, q (6.7)	32.4	1.96, q (6.5)
7	32.1	2.12, m	32.2	2.11, m
8	131.4	5.42, dt (15.2, 6.3)	131.3	5.41, m
9	129.0	5.37, dt (15.2, 6.4)	129.0	5.38, m
10	32.6	2.08, m	32.2	2.07, m
11~15	29.2–29.7	1.26, br s	29.2–29.7	1.26, br s
16	31.9	1.26, br s	32.0	1.26, br s
17	22.7	1.26, br s	22.7	1.26, br s
18	14.1	0.88, t (6.8)	14.1	0.87, t (7.0)
1′	174.0	–	175.0	–
2′	36.9	2.23, t (7.5)	72.4	4.12, dd (7.8, 3.4)
3′	25.8	1.64, m	34.8	2.16, t (3.3)
4′	29.2–29.7	1.26, br s	31.8	1.26, br s
5′	29.2–29.7	1.26, br s	25.1	1.63, m
6′~11′	29.2–29.7	1.26, br s	29.2–29.7	1.26, br s
12′	31.9	1.26, br s	31.9	1.26, br s
13′	22.7	1.26, br s	22.7	1.26, br s
14′	14.1	0.88, t (6.8)	14.1	0.87, t (7.0)
NH	–	6.25, d (7.5)	–	6.20, d (7.8)

^a^ Assignments based on DEPTs and HSQC.

**Table 3:** Cytotoxicity of compounds **1**–**9** against HL-60, HepG2 and SGC7901 tumor cell lines in vitro (IC_50_, μM) **^a^**.

Compound	HL-60	HepG-2	SGC-7901
1	17.64 ± 0.32	12.34 ± 0.12	18.37 ± 0.17
2	>100	>100	>100
3	>100	>100	>100
4	>100	>100	>100
5	6.29 ± 0.11	4.12 ± 0.15	7.32 ± 0.26
6	32.26 ± 0.23	26.69 ± 0.21	27.14 ± 0.30
7	25.32 ± 0.17	21.13 ± 0.13	22.74 ± 0.16
8	35.72 ± 0.36	28.53 ± 0.24	30.31 ± 0.14
9	58.15 ± 0.28	46.21 ± 0.17	45.79 ± 0.12
Adriamycin ^b^	2.51 ± 0.14	2.73 ± 0.23	2.65 ± 0.17

^a^ IC_50_ values are means from three independent experiments in which each compound concentration was tested in three replicate wells; ^b^ Adriamycin as a positive control.

## Supplementary Materials

The following are available online at www.mdpi.com/1660-3397/15/4/120/s1, Figures S1–S9: HR-ESI-MS, ESI-MS, ^1^H-NMR, ^13^C-NMR, DEPT135, ^1^H-^1^H COSY, HSQC, HMBC and NOESY spectra for new compound **1**, Figures S10, S12 and S13: HR-ESI-MS, ^1^H-NMR and ^13^C-NMR spectra for new compound **6**, Figures S14, S16 and S17: HR-ESI-MS, ^1^H-NMR and ^13^C-NMR spectra for new compound **7**, Figures S11 and S15: EI-MS spectra of methyl tetradecanoate and (*R*)-methyl 2-hydroxytetradecanoate obtained from methanolysis of ceramides **6** and **7**, respectively, Data S18–S22: ^1^H-NMR and ^13^C-NMR data for the known compounds **2**–**5**, Data S23 and S24: ^1^H-NMR, ^13^C-NMR and ESI-MS data for the known compounds **8** and **9**.

## Abbreviations

The following abbreviations are used in this manuscript:

CC	Column Chromatography
HPLC	High-Performance Liquid Chromatography
MTT	Microculture Tetrazolium
ATCC	American Type Culture Collection
RPMI	Roswell Park Memorial Institute
LCB	Long Chain Base
FAB	Fatty Acid Base
TD-DFT	Time-Dependent Density Functional Theory
