# Supplementary material for: New Cytotoxic Secondary Metabolites from Marine Bryozoan Cryptosula pallasiana
Source: Mar Drugs. 2017 Apr 13;15(4):120. doi: 10.3390/md15040120 (PMC5408266; doi:10.3390/md15040120)
Supplement: Supplementary file 1 [file marinedrugs-15-00120-s001.pdf]

New Cytotoxic Secondary Metabolites from Marine Bryozoan *Cryptosula pallasiana*

Xiang-Rong Tian [1, 2†], Yan-Qing Gao [1,†], Xiao-Lin Tian [1], Hai-Feng Tang [2,*], Yu-Shan Li [3], Hou-Wen Lin [4] and Zhi-Qing Ma [1,*]

* Author to whom correspondence should be addressed; tanghaifeng71@163.com; zhiqingma@nwsuaf.edu.cn (Z.-Q. M.); Tel./Fax.: +86-29-84774748; Tel.: +86-29-87092122; Fax.: +86-29-87093344

† These authors contributed equally to this work.

Supplementary Information

S1. HR-ESI-MS (positive) spectrum of compound **1**

S2. ESI-MS (positive) spectrum of compound **1**

S3. ^{1}H-NMR ($CDCl_3$, 500 MHz) spectrum of compound **1**

S4. ^{13}C-NMR ($CDCl_3$, 125 MHz) spectrum of compound **1**

S5. DEPT135 spectrum of compound **1**

S6. ^{1}H-^{1}H COSY spectrum of compound **1**

S7. HSQC spectrum of compound **1**

S8. HMBC spectrum of compound **1**

S9. NOESY spectrum of compound **1**

S10. HR-ESI-MS (positive) spectrum of compound **6**

S11. EI-MS spectrum of methyl tetradecanoate obtained from methanolysis of ceramide **6**

S12. ^{1}H-NMR ($CDCl_3$, 500 MHz) spectrum of compound **6**

S13. ^{13}C-NMR ($CDCl_3$, 125 MHz) spectrum of compound **6**

S14. HR-ESI-MS (positive) spectrum of compound **7**

S15. EI-MS spectrum of (*R*)-methyl 2-hydroxytetradecanoate obtained from methanolysis of ceramide **7**

S16. ^{1}H-NMR ($CDCl_3$, 500 MHz) spectrum of compound **7**

S17. ^{13}C-NMR ($CDCl_3$, 125 MHz) spectrum of compound **7**

S18. ^{1}H-NMR ($CDCl_3$, 500 MHz) data of compound **2**

S19. ^{1}H-NMR ($CDCl_3$, 500 MHz) data of compound **3**

S20. ^{1}H-NMR ($CDCl_3$, 500 MHz) data of compound **4**

S21. ^{13}C-NMR ($CDCl_3$, 125 MHz) data of compounds **2–4**

S22. ^{1}H-NMR (CD_3OD, 500 MHz) and ^{13}C NMR (CD_3OD, 125 MHz) data of compound **5**

S23. ^{1}H-NMR ($CDCl_3$, 500MHz), ^{13}C-NMR ($CDCl_3$, 125 MHz) and ESI-MS data of compound **8**

S24. ^{1}H-NMR ($CDCl_3$, 500MHz), ^{13}C-NMR ($CDCl_3$, 125 MHz) and ESI-MS data of compound **9**

S1. HR-ESI-MS (positive) spectrum of compound **1**

Elemental Composition Report **Page 1**

Multiple Mass Analysis: 2 mass(es) processed
Tolerance = 30.0 PPM / DBE: min = -1.5, max = 50.0
Selected filters: None

Monoisotopic Mass, Even Electron Ions
16 formula(e) evaluated with 2 results within limits (up to 50 closest results for each mass)
Elements Used:
C: 5-34 H: 10-55 O: 1-2 Na: 1-1

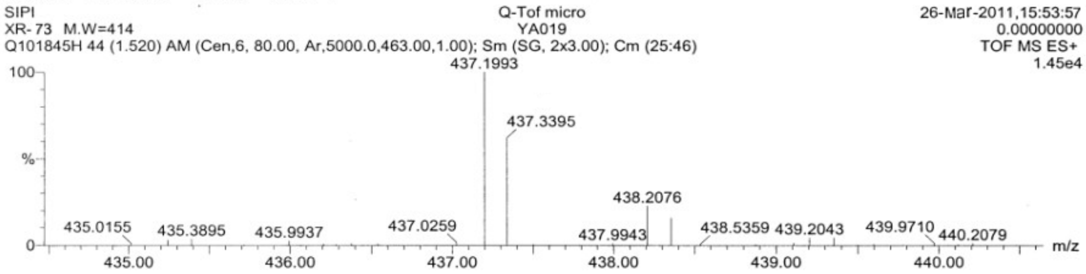

Minimum:	45.00				-1.5		
Maximum:	100.00		5.0	30.0	50.0		
Mass	RA	Calc. Mass	mDa	PPM	DBE	i-FIT	Formula
437.1993	100.00	437.1881	11.5	26.3	18.5	378.3	C31 H26 O Na
437.3395	61.83	437.3396	-0.2	-0.5	5.5	79.2	C28 H46 O2 Na

S2. ESI-MS (positive) spectrum of compound **1**

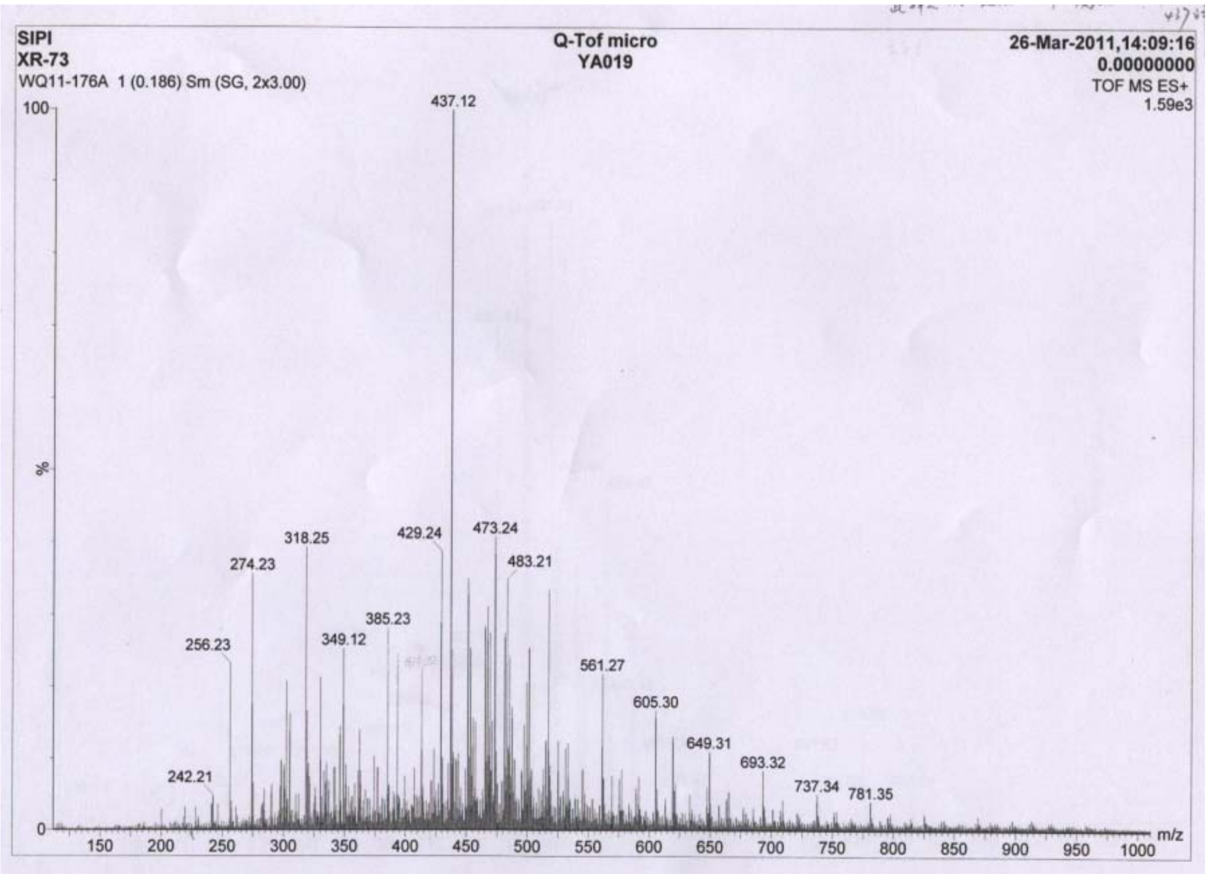

S3. ^{1}H-NMR ($CDCl_3$, 500 MHz) spectrum of compound **1**

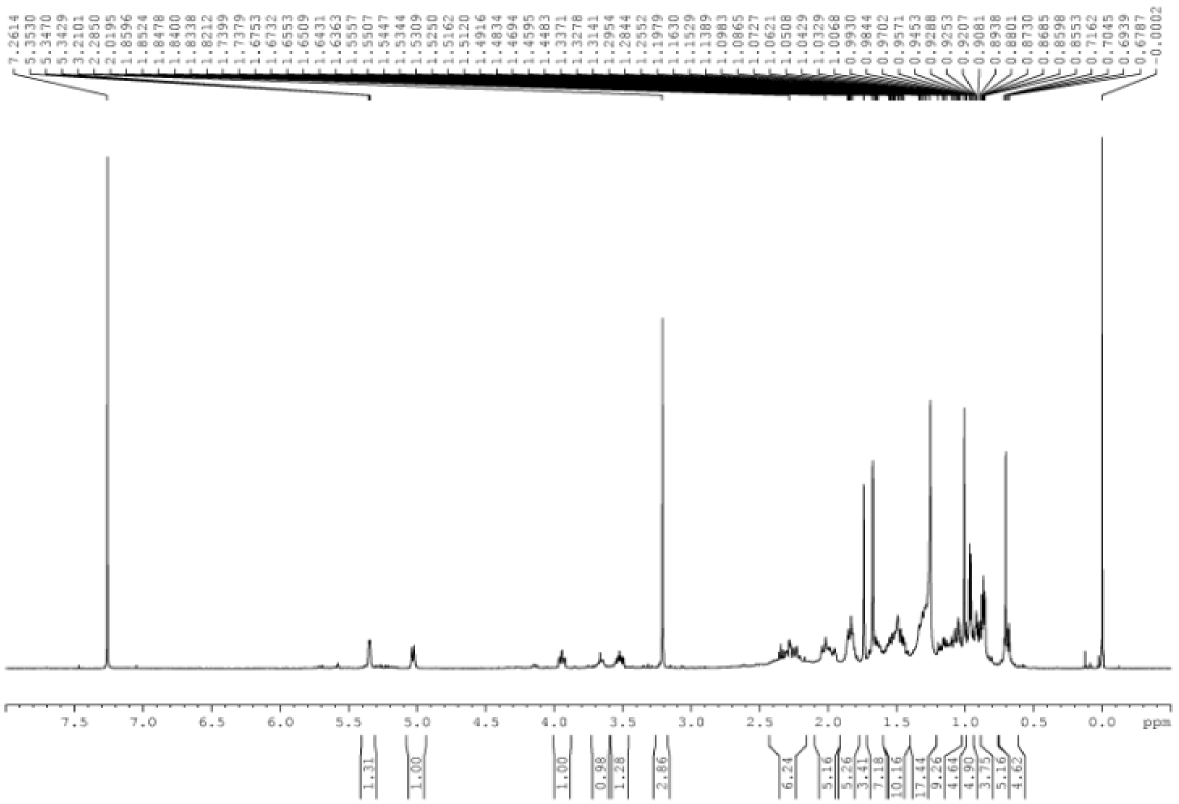

S4. ^{13}C NMR ($CDCl_3$, 125 MHz) spectrum of compound **1**

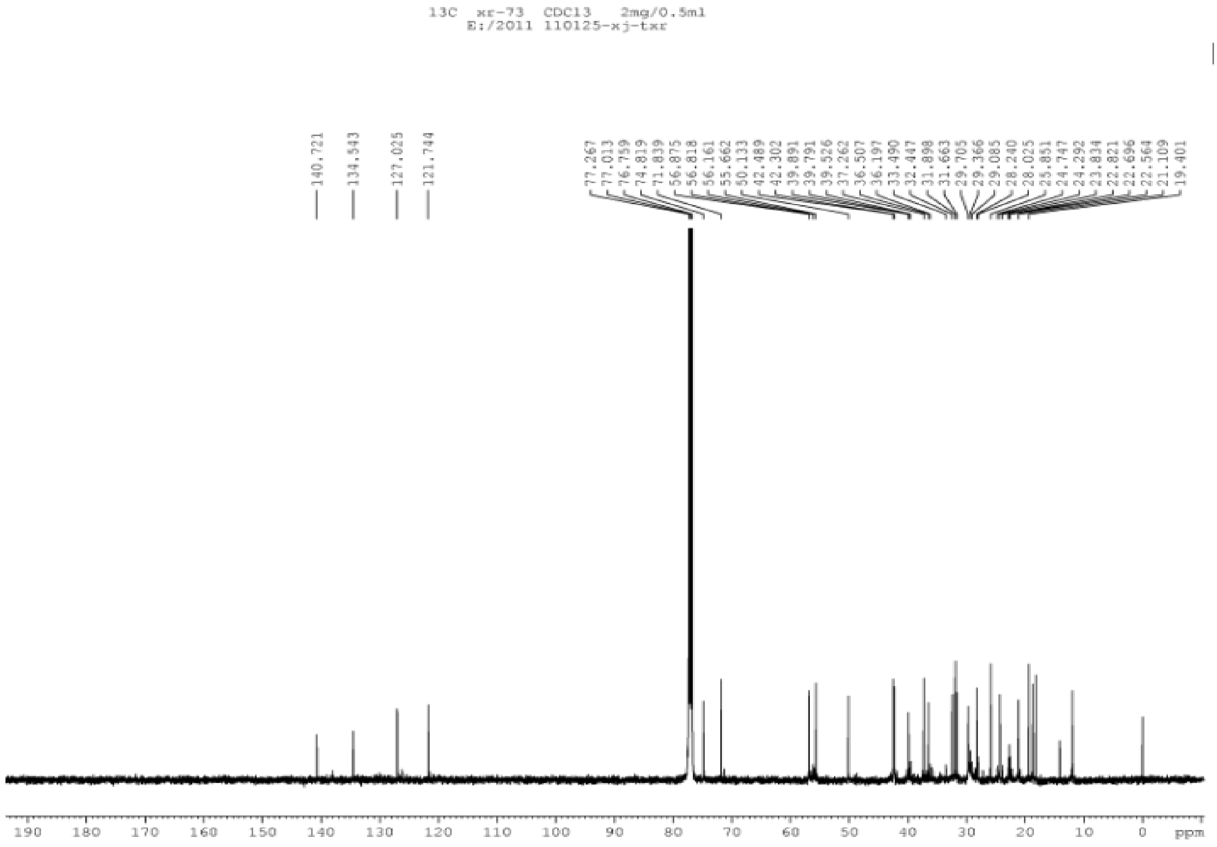

S5. DEPT135 spectrum of compound **1**

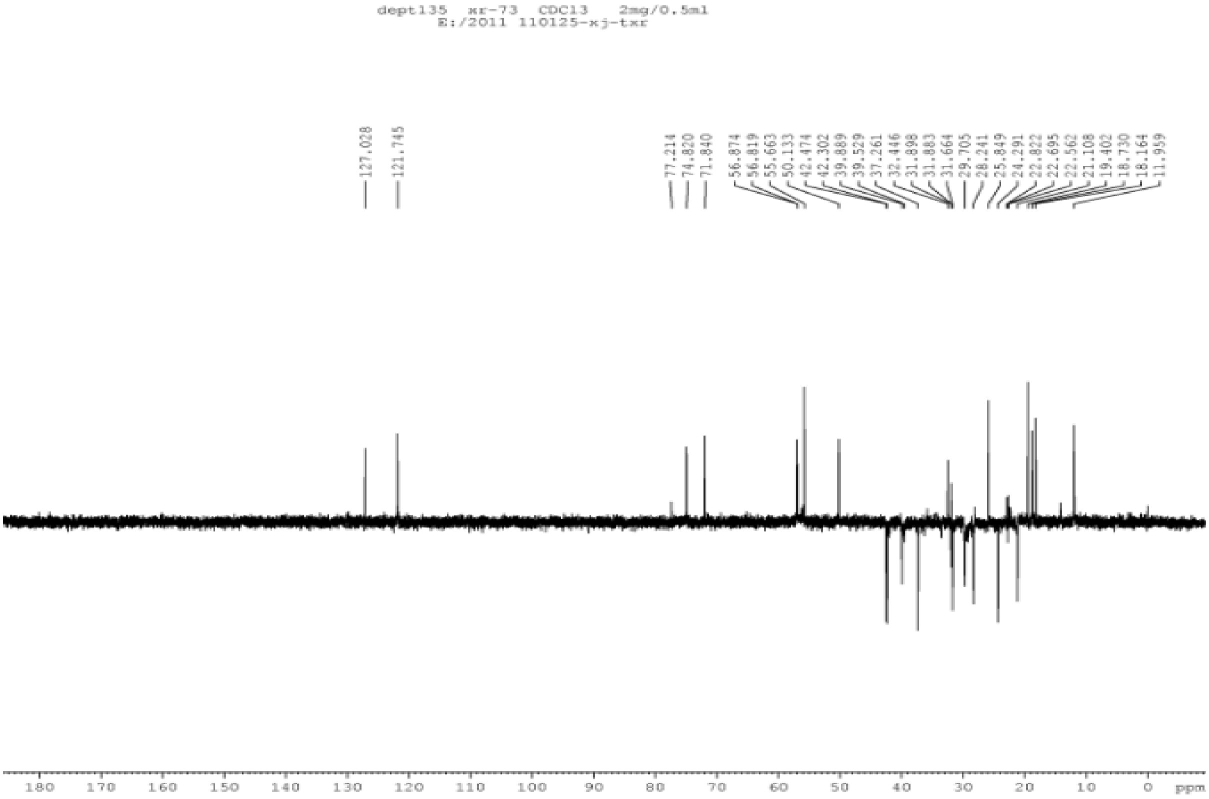

S6. ^{1}H-^{1}H COSY spectrum of compound **1**

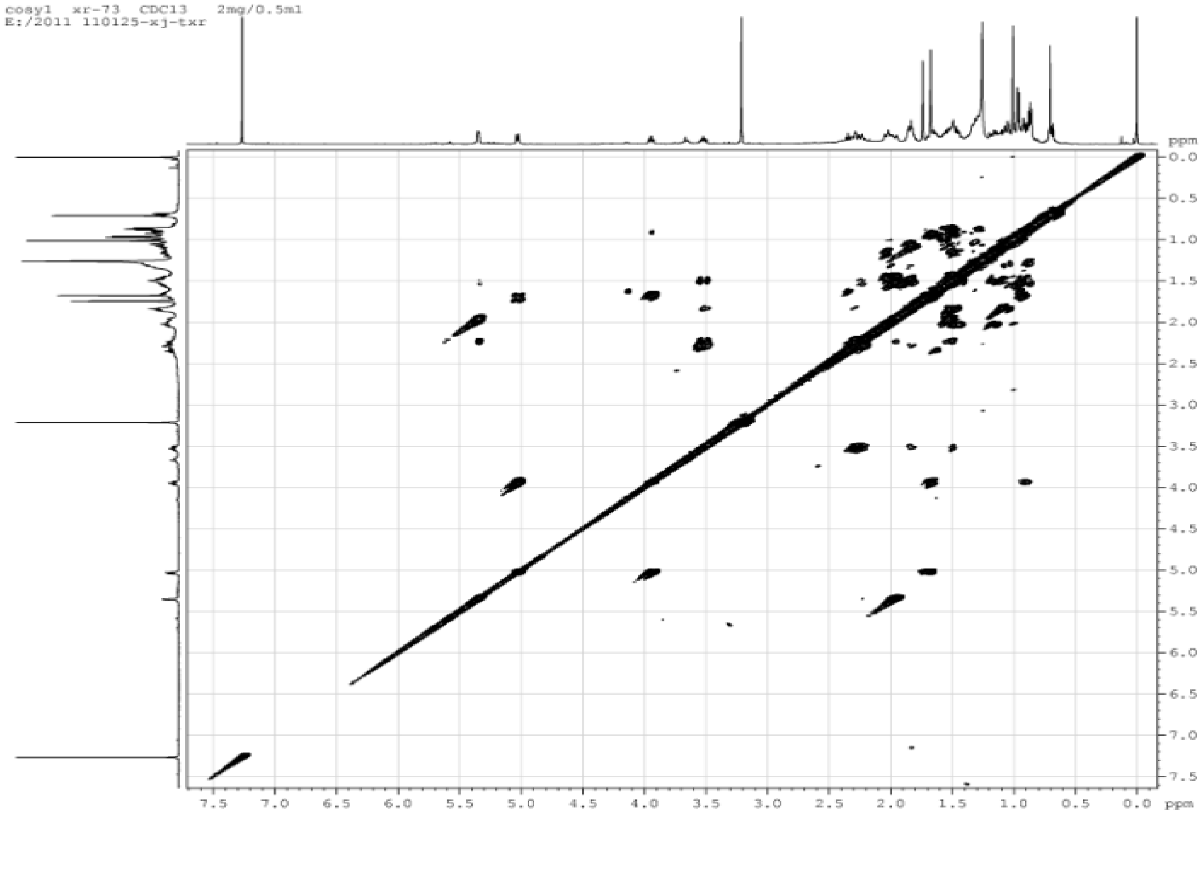

S7. HSQC spectrum of compound **1**

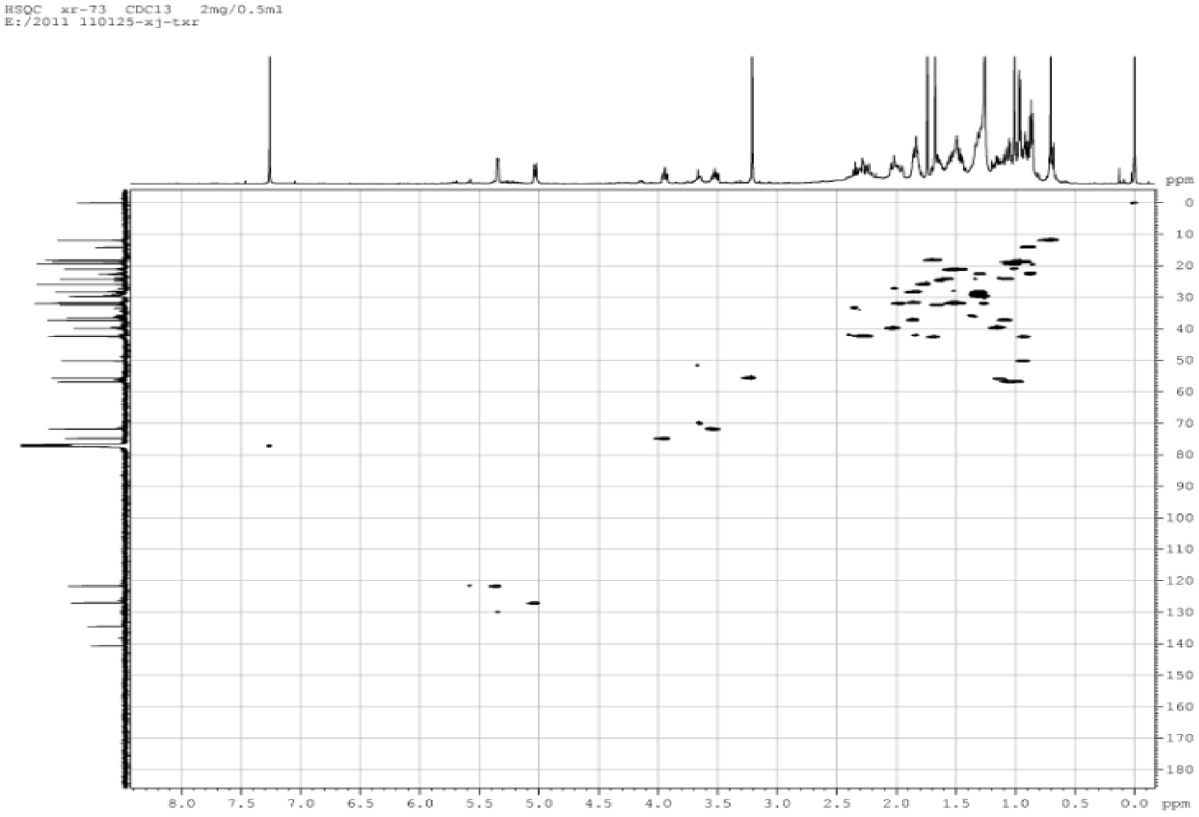

S8. HMBC spectrum of compound **1**

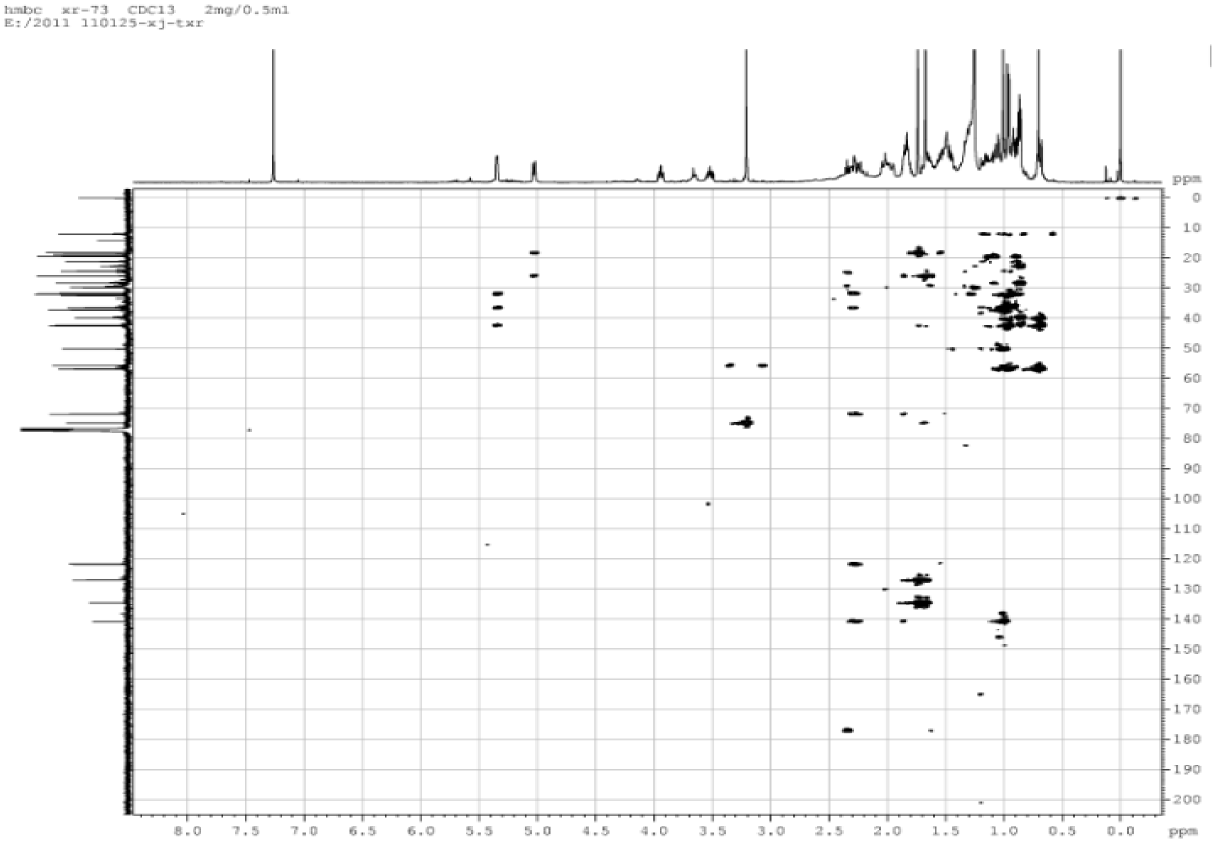

S9. NOESY spectrum of compound **1**

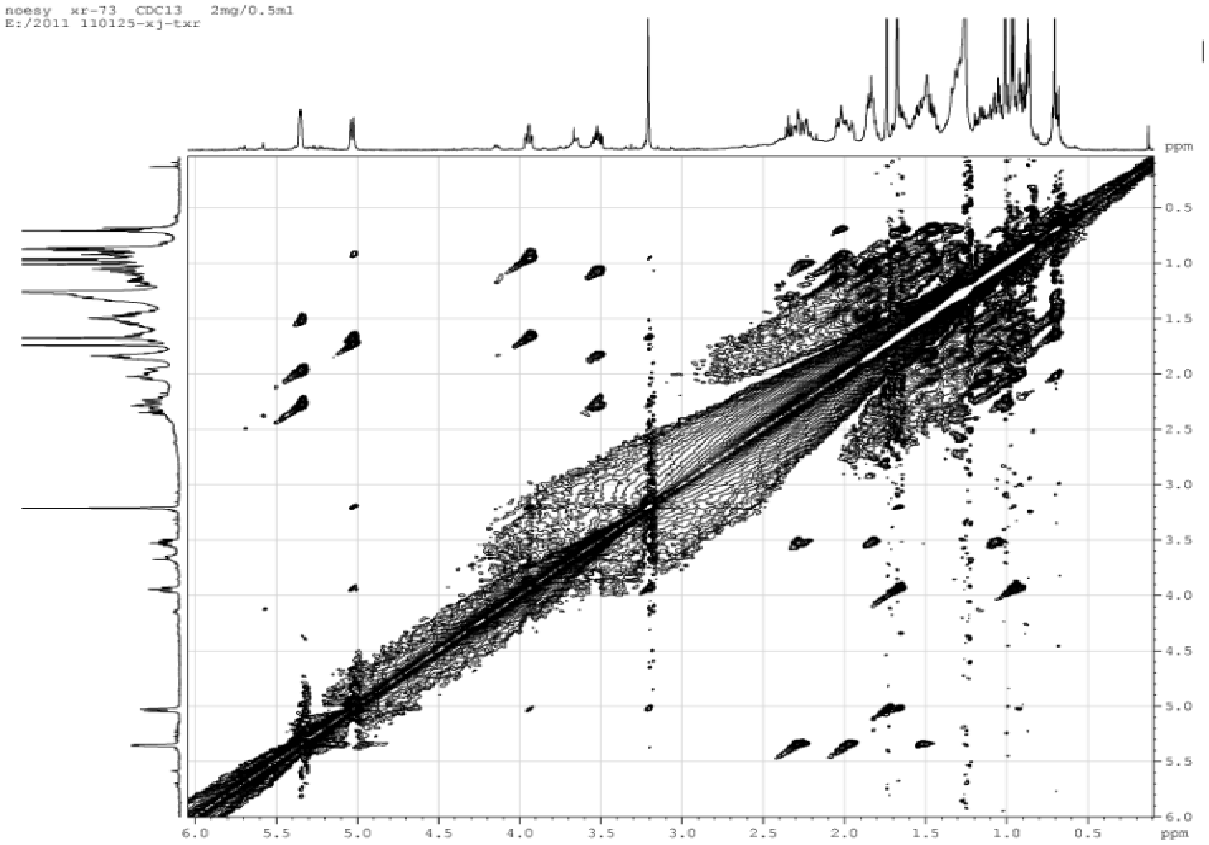

S10. HR-ESI-MS (positive) spectrum of compound **6**

Elemental Composition Report **Page 1**

Multiple Mass Analysis: 2 mass(es) processed
Tolerance =1 0.0 PPM / DBE: min = -1.5, max = 50.0
Selected filters: None

Monoisotopic Mass, Even Electron Ions
16 formula(e) evaluated with 2 results within limits (up to 50 closest results for each mass)
Elements Used:
C:10-35 H:5-57 N:1-1 O:1-6 Na:1-1

SIPI Q-Tof micro 26-Mar-2011,15:57:53
XR-70 M.W=507 YA019 0.00000000
Q101845H 44 (1.520) AM (Cen,6, 80.00, Ar,5000.0,463.00,1.00); Sm (SG, 2x3.00); Cm (25:46) TOF MS ES+ 5.45e4

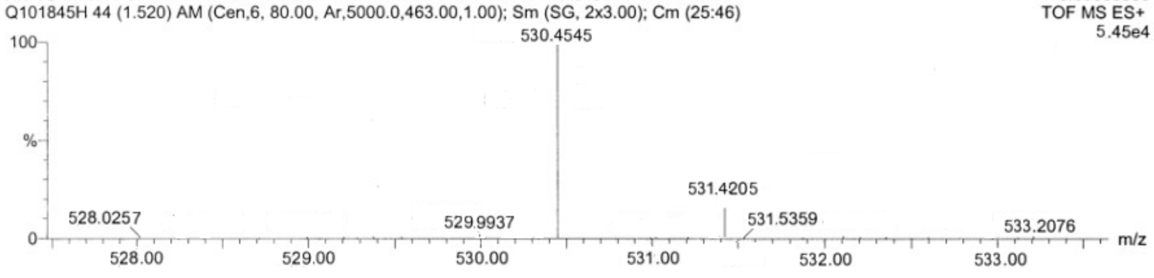

Minimum:	45.00				-1.5		
Maximum:	100.00		5.0	30.0	50.0		
Mass	RA	Calc. Mass	mDa	PPM	DBE	i-FIT	Formula
530.4545	100.00	530.4549	-0.4	6.5	18.3	38.5	C32 H61 N O3 Na

S11. EI-MS spectrum of methyl tetradecanoate obtained from methanolysis of ceramide **6**

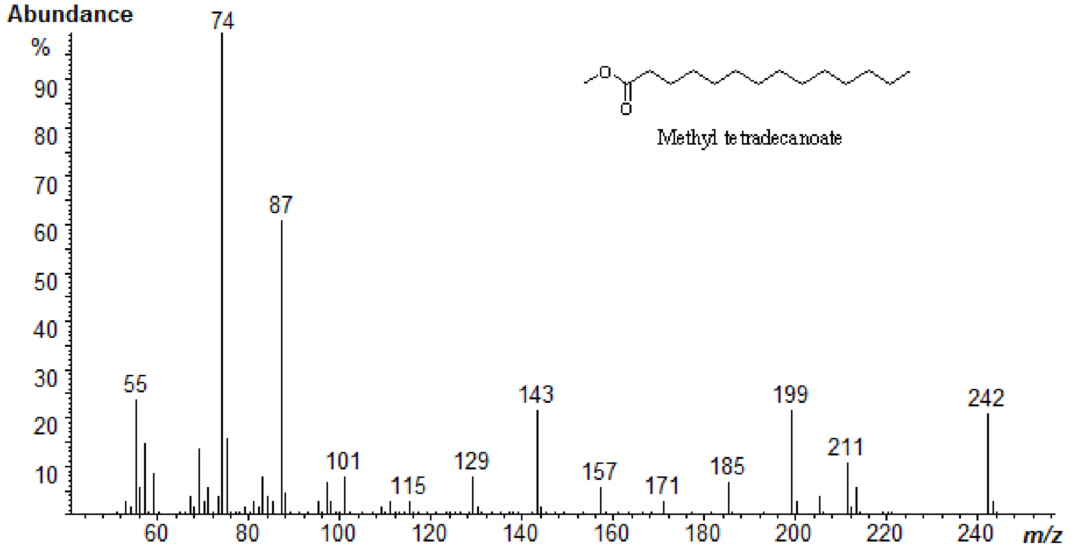

S12. ^{1}H-NMR ($CDCl_3$, 500 MHz) spectrum of compound **6**

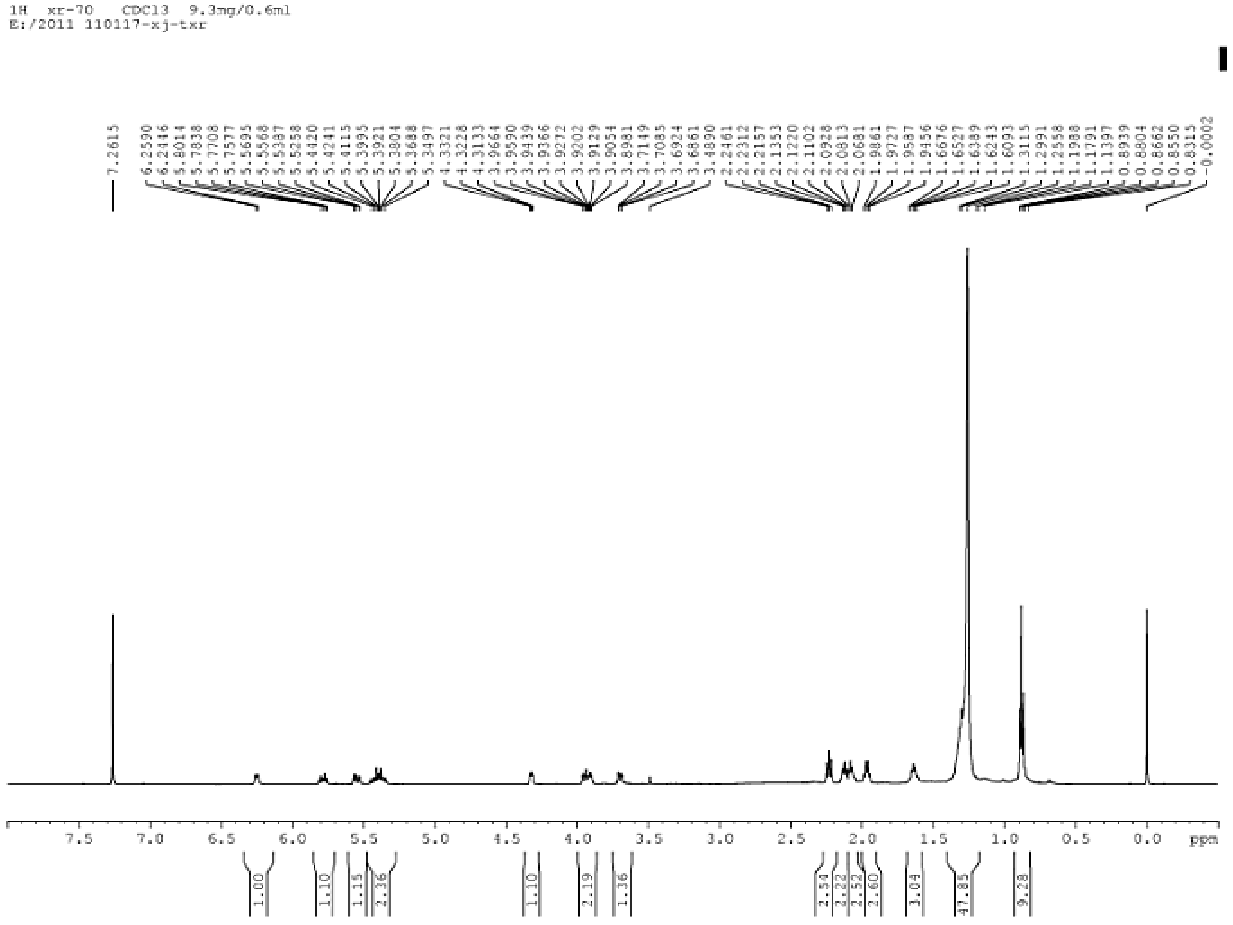

S13. ^{13}C-NMR ($CDCl_3$, 125 MHz) spectrum of compound **6**

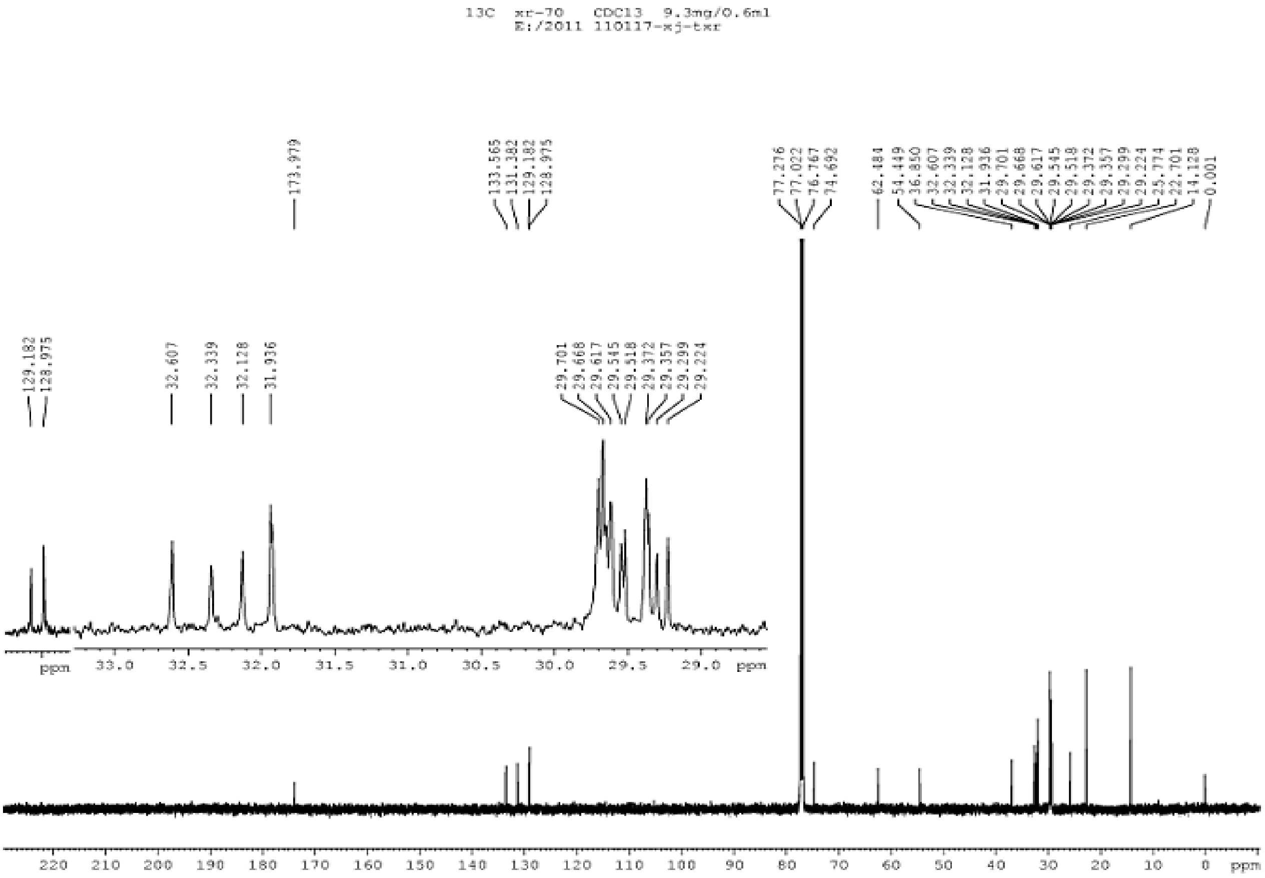

S14. HR-ESI-MS (positive) spectrum of compound **7**

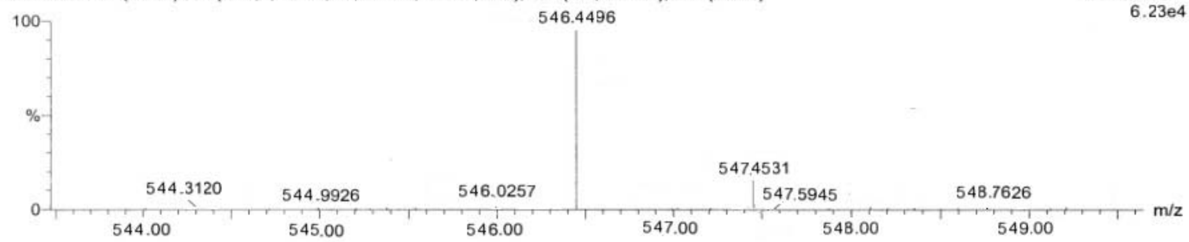

Elemental Composition Report **Page 1**

Multiple Mass Analysis: 2 mass(es) processed
Tolerance = 1 0.0 PPM / DBE: min = -1.5, max = 50.0
Selected filters: None

Monoisotopic Mass, Even Electron Ions
16 formula(e) evaluated with 2 results within limits (up to 50 closest results for each mass)
Elements Used:
C:10-35 H:5-57 N:1-1 O:1-6 Na:1-1

SIPI Q-Tof micro 26-Mar-2011,16:25:53
XR-71 M.W= 523 YA019 0.00000000
Q101845H 44 (1.520) AM (Cen,6, 80.00, Ar,5000.0,463.00,1.00); Sm (SG, 2x3.00); Cm (25:46) TOF MS ES+ 6.23e4

Minimum:	45.00				-1.5		
Maximum:	100.00		5.0	30.0	50.0		
Mass	RA	Calc. Mass	mDa	PPM	DBE	i-FIT	Formula
546.4496	100.00	546.4498	-0.2	4.5	16.8	30.5	C32 H61 N O4 Na

S15. EI-MS spectrum of (R)-Methyl 2-hydroxytetradecanoate obtained from methanolysis of ceramide **7**

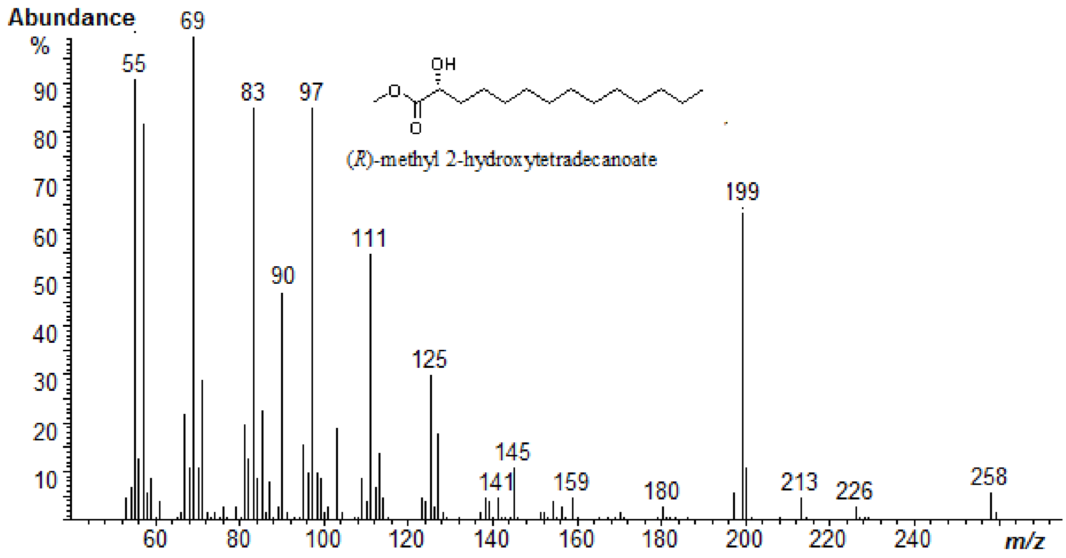

S16. ^{1}H-NMR ($CDCl_3$, 500 MHz) spectrum of compound **7**

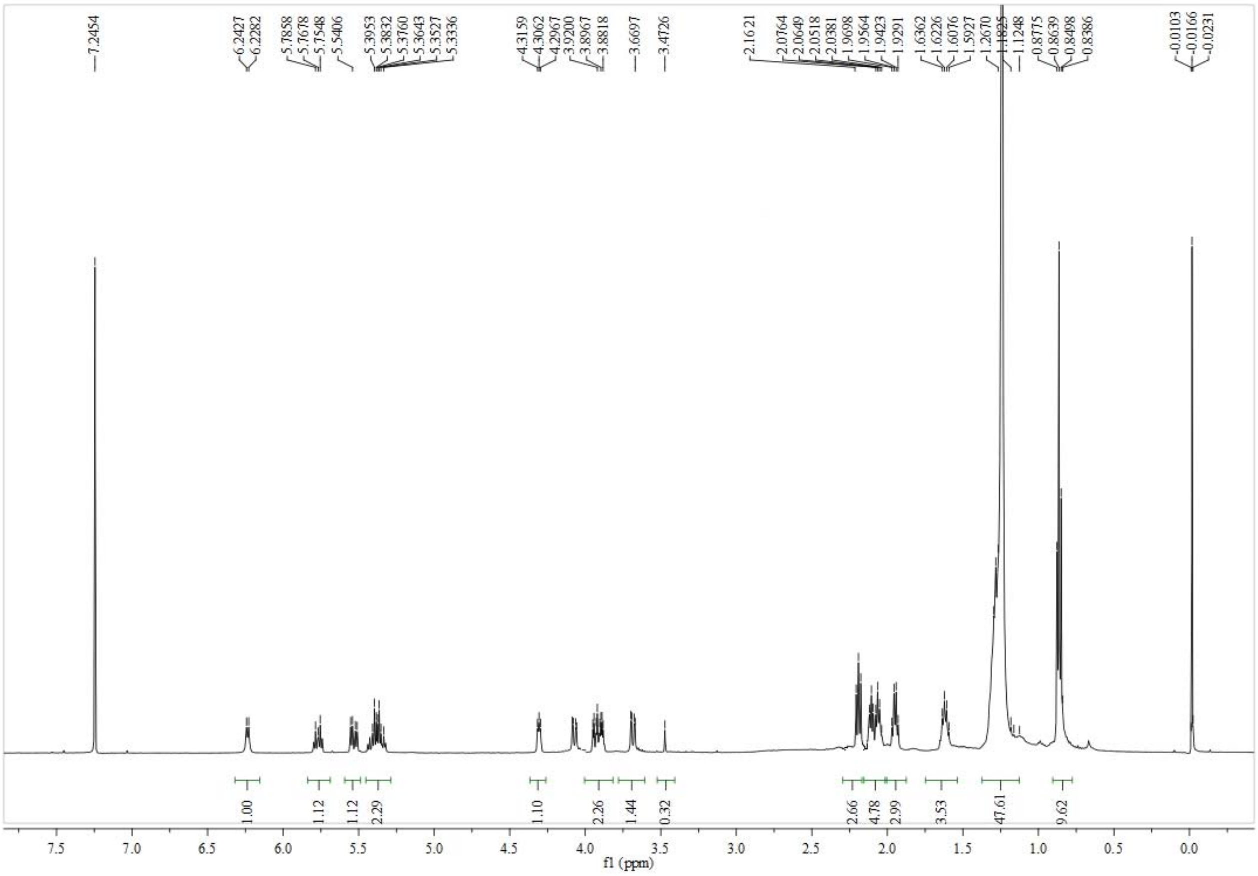

S17. ^{13}C-NMR ($CDCl_3$, 125 MHz) spectrum of compound **7**

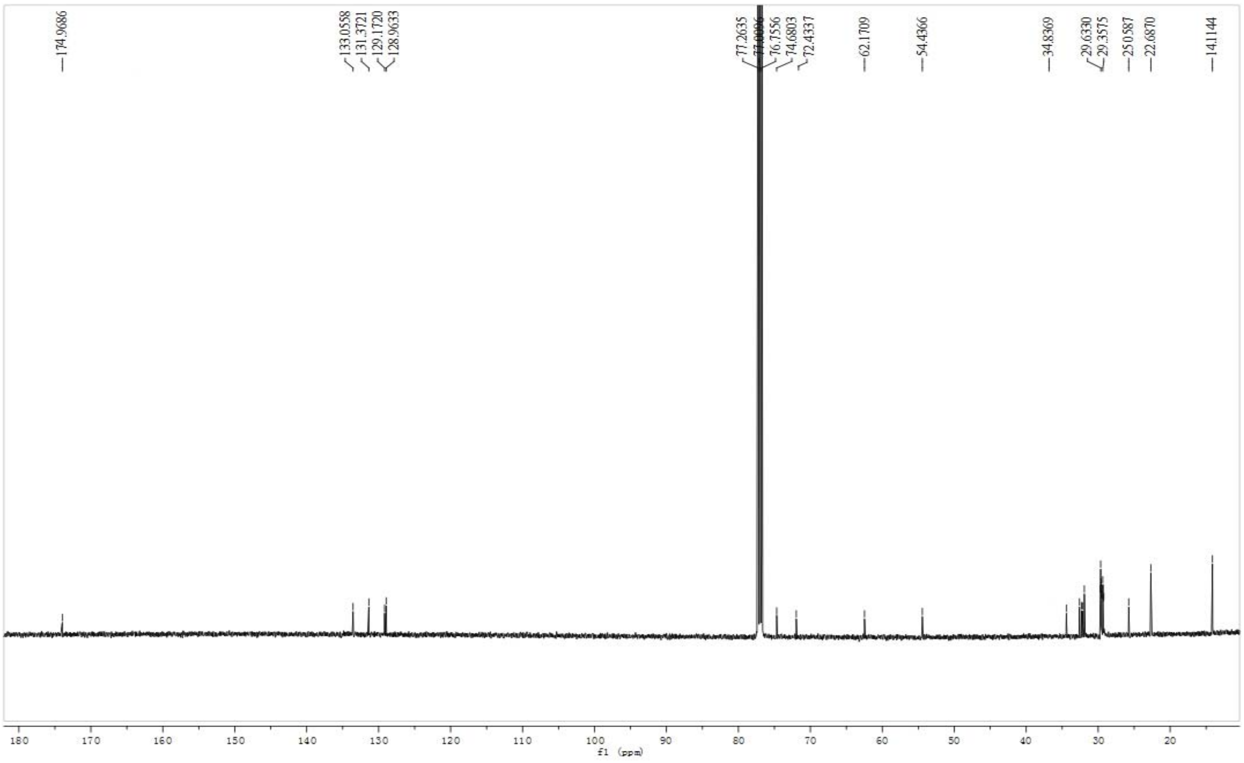

S18. ^{1}H-NMR ($CDCl_3$, 500MHz) data of compound **2**

^{1}H-NMR (500MHz, $CDCl_3$) δ: 5.35 (1H, *m*, H-6), 3.52 (1H, *m*, H-3), 1.00 (3H, *s*, H_3-19), 0.91 (3H, *d*, J = 6.5 Hz, H_3-21), 0.87 (3H, *d*, J = 6.5 Hz, H_3-26), 0.86 (3H, *d*, J = 6.5 Hz, H_3-27), 0.68 (3H, *s*, H_3-18).

S19. ^{1}H-NMR ($CDCl_3$, 500MHz) data of compound **3**

^{1}H NMR (500 MHz, $CDCl_3$) δ: 5.35 (1H, *t*, J = 2.6 Hz, H-7), 5.28 (1H, *dd*, J = 15.3, 6.7 Hz, H-23), 5.20 (1H, *dd*, J = 15.5, 7.0 Hz, H-22), 4.08 (1H, *m*, H-3), 3.63 (1H, *d*, J = 5.1 Hz, H-6), 1.18 (3H, *s*, H_3-19), 1.00 (3H, *d*, J = 6.6 Hz, H_3-21), 0.86 (6H, *d*, J = 6.6 Hz, H_3-26, H_3-27), 0.69 (3H, *s*, H-18).

S20. ^{1}H-NMR ($CDCl_3$, 500MHz) data of compound **4**

^{1}H-NMR (500 MHz, $CDCl_3$) δ: 5.35 (1H, *m*, H-7), 5.21 (1H, *dd*, J = 15.2, 7.5 Hz, H-23), 5.16 (1H, *dd*, J = 15.1, 7.4 Hz, H-22), 4.07 (1H, *m*, H-3), 3.63 (1H, *d*, J = 5.1 Hz, H-6), 1.09 (3H, *s*, H_3-19), 1.02 (3H, *d*, J = 6.7 Hz, H_3-21), 0.92 (3H, *d*, J = 6.8 Hz, H_3-28), 0.84 (3H, *d*, J = 6.4 Hz, H_3-26), 0.82 (3H, *d*, J = 6.5 Hz, H_3-27), 0.60 (3H, *s*, H_3-18).

S21. ^{13}C-NMR ($CDCl_3$, 125 MHz) data of compounds **2 – 4** †

	**2**	**3**	**4**
**NO.**	δ_C (*mult.*)	δ_C (*mult.*)	δ_C (*mult.*)
1	37.3 *t*	33.0 *t*	33.0 *t*
2	31.7 *t*	30.8 *t*	30.9 *t*
3	71.8 *d*	67.8 *d*	67.8 *d*
4	42.3 *t*	39.5 *t*	39.2 *t*
5	140.8 *s*	76.1 *s*	76.1 *s*
6	121.7 *d*	73.7 *d*	73.7 *d*
7	31.9 *t*	117.5 *d*	117.5 *d*
8	31.9 *d*	144.1 *s*	144.0 *s*
9	50.1 *d*	43.5 *d*	43.5 *d*
10	36.5 *s*	37.1 *s*	37.2 *s*
11	21.1 *t*	22.1 *t*	22.1 *t*
12	39.8 *t*	39.8 *t*	39.5 *t*
13	42.3 *s*	43.8 *s*	43.8 *s*
14	56.8 *d*	54.7 *d*	54.8 *d*
15	24.3 *t*	22.9 *t*	22.9 *t*
16	28.2 *t*	28.6 *t*	28.2 *t*
17	56.2 *d*	56.0 *d*	55.9 *d*
18	11.9 *q*	12.3 *q*	12.3 *q*
19	19.4 *q*	18.8 *q*	18.8 *q*
20	35.8 *d*	40.2 *d*	40.5 *d*
21	18.7 *q*	20.8 *q*	21.1 *q*
22	36.2 *t*	138.1 *d*	135.6 *d*
23	23.8 *t*	126.3 *d*	132.3 *d*
24	39.5 *t*	42.0 *t*	43.1 *d*
25	28.3 *d*	28.5 *d*	33.2 *d*
26	22.8 *q*	22.2 *q*	19.7 *q*
27	22.6 *q*	22.3 *q*	20.2 *q*
28			18.0 *q*

† Assignments aided by DEPT experiments and literatures.

S22. ^{1}H-NMR (CD_3OD, 500 MHz) and ^{13}C-NMR (CD_3OD, 125 MHz) data of compound **5**

^{1}H-NMR (CD_3OD, 500 MHz) δ: 5.74 (1H, *s*, H-7), 4.21 (1H, *tt*, *J* = 7.0, 3.7 Hz, H-3), 2.41 (1H, *dt*, *J* = 13.9, 2.5 Hz, H-4b), 1.98 (1H, *dt*, *J* = 14.4, 2.5 Hz, H-2b), 1.75 (3H, *s*, H_3-11), 1.72 (1H, *d*, *J* =

4.1 Hz, H-4a), 1.51 (1H, *dd*, *J* = 14.4, 5.0 Hz, H-2a), 1.46 (3H, *s*, H_3-10), 1.26 (3H, *s*, H_3-9); ^{13}C-NMR (CD_3OD, 125 MHz) δ: 185.7 (*s*, C-6), 174.4 (*s*, C-8), 113.3 (*d*, C-7), 89.0 (*s*, C-5), 67.2 (*d*, C-3), 48.0 (*t*, C-2), 46.4 (*t*, C-4), 37.2 (*s*, C-1), 31.0 (*q*, C-9), 27.4 (*q*, C-11), 27.0 (*q*, C-10).

S23. ^{1}H-NMR ($CDCl_3$, 500MHz), ^{13}C-NMR ($CDCl_3$, 125 MHz) and ESI-MS data of compound **8**

^{1}H-NMR (500 MHz, $CDCl_3$) δ: 6.25 (1H, *d*, *J* = 7.2 Hz, NH), 5.78 (1H, *dt*, *J* = 15.0, 6.6 Hz, H-5), 5.53 (1H, *dd*, *J* = 15.3, 6.5 Hz, H-4), 4.32 (1H, *br t*, *J* = 4.4 Hz, H-3), 3.95 (1H, *dd*, *J* = 11.2, 3.7 Hz, H-2), 3.90 (1H, *m*, H-1b), 3.70 (1H, *dd*, *J* = 11.2, 3.2 Hz, H-1a), 2.23 (2H, *t*, *J* = 7.5 Hz, H-2′), 2.05 (4H, *q*, J = 7.1, H-6), 1.63 (2H, m, H-3′), 1.26-1.37 (42H, *m*, H-7 − H-17, H-4′ – H-13′), 0.88 (6H, *t*, *J* = 7.0 Hz, H-18, H-14′); ^{13}C-NMR (125 MHz, $CDCl_3$) δ: 174.0 (C-1′), 134.3 (C-5), 128.8 (C-4), 74.7 (C-3), 62.5 (C-1), 54.5 (C-2), 36.9 (C-2′), 32.3 (C-6), 31.9 (C-12′, C-7, C-16), 29.1-29.7 (C-8 – C-15, C-4′ − C-11′), 25.8 (C-3′), 22.7 (C-17, C-13′), 14.1 (C-18, C-14′); ESI-MS (+) *m/z*: 532 [M + Na]$^+$, 437, 330, 302, 274, 264, 256, 219; ESI-MS (–) *m/z*: 554, [M + COOH]$^-$, 544 [M + Cl]$^-$, 508 [M – H]$^-$, 447, 394, 339, 325, 311, 283, 265, 255, 227, 143, 126.

S24. ^{1}H-NMR ($CDCl_3$, 500MHz), ^{13}C-NMR ($CDCl_3$, 125 MHz) and ESI-MS data of compound **9**

^{1}H-NMR (500 MHz, $CDCl_3$) δ: 6.25 (1H, *d*, *J* = 7.5 Hz, NH), 5.77 (1H, *dt*, *J* = 15.2, 6.65 Hz, H-5), 5.54 (1H, *dd*, *J* = 15.4, 6.4 Hz, H-4), 5.42 (1H, *dt*, *J* = 15.2, 6.3 Hz, H-9), 5.37 (1H, *dt*, *J* = 15.2, 6.4 Hz, H-8), 4.32 (1H, *br s*, H-3), 3.94 (1H, *dd*, *J* = 11.3, 3.7 Hz, H-2), 3.91 (1H, *m*, H-1b), 3.71 (1H, *dd*, *J* = 11.3, 3.5 Hz, H-1a), 2.23 (2H, *t*, *J* = 7.5 Hz, H-2′), 2.12 (2H, *m*, H-7), 2.07 (2H, *m*, H-10), 1.96 (2H, *q*-like, *J* = 6.7 Hz, H-6), 1.64 (2H, m, H-3′), 1.26 (38H, *m*, H-11 − H-17, H-4′ – H-15′), 0.88 (6H, *t*, *J* = 6.8 Hz, H-18, H-16′); ^{13}C-NMR (125 MHz, $CDCl_3$) δ: 174.0 (C-1′), 133.6 (C-5), 131.4 (C-8), 129.2 (C-4), 129.0 (C-9), 74.7 (C-3), 62.5 (C-1), 54.5 (C-2), 36.9 (C-2′), 32.6 (C-10), 32.3 (C-6), 32.1 (C-7), 31.9 (C-14′, C-16), 29.2-29.7 (C-11 – C-15, C-4′ − C-13′), 25.8 (C-3′), 22.7 (C-17, C-15′), 14.1 (C-18, C-16′); ESI-MS (+) *m/z*: 558 [M + Na]$^+$, 437, 330, 274, 262, 256; ESI-MS (–) *m/z*: 570 [M + Cl]$^-$, 534 [M – H]$^-$, 325, 311, 283, 255, 155, 113.
